# *rac*-*N*-(4-Eth­oxy­phen­yl)-3-hy­droxy­butanamide

**DOI:** 10.1107/S2414314623002316

**Published:** 2023-03-15

**Authors:** James E. Hines III, Zechariah Myles, Garrick Breaux, Frank R. Fronczek, Rao M. Uppu

**Affiliations:** a Department of Environmental Toxicology, Southern University and A&M College, Baton Rouge, LA 70813, USA; b Department of Chemistry, Louisiana State University, Baton Rouge, LA 70803, USA; Katholieke Universiteit Leuven, Belgium

**Keywords:** bucetin, non-opioid analgesics, crystal structure, hydrogen bonding

## Abstract

In the title compound, racemic bucetin [systematic name: *N*-(4-eth­oxy­phen­yl)-3-hydroxy­butanamide], C_12_H_17_NO_3_, the mol­ecule is in an extended conformation as illustrated by the C—O—C—C torsion angle [170.14 (15)°] in the eth­oxy group and the subsequent C—N—C—C [−177.24 (16)°], N—C—C—C [170.08 (15)°] and C—C—C—C [171.41 (15)°] torsion angles in the butanamide chain. In the crystal, the O—H group donates an inter­molecular O—H⋯O hydrogen bond to the amide carbonyl oxygen atom and also accepts an inter­molecular N—H⋯O hydrogen bond from an adjacent N—H group. The former forms 12-membered dimeric rings about inversion centers, and the latter form chains in the [001] direction. The overall hydrogen-bonded network is two-dimensional, with no propagation in the [100] direction.

## Abstract 2

The crystal structure of bucetin, an analgesic and anti­pyric similar to phenacetin, is presented.

## Graphical abstract

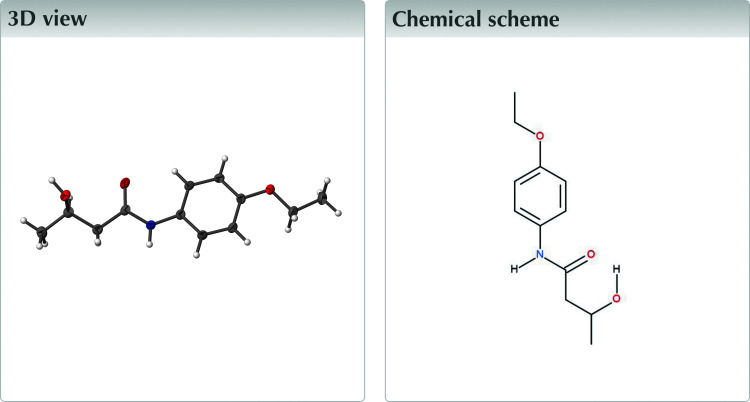

## Structure description

*N*-(4-Eth­oxy­phen­yl)-3-hydro­butanamide, popularly known as bucetin, is an analgesic and anti­pyric that is similar in structure to phenacetin [*N*-(4-eth­oxy­phen­yl)acetamide]. Once thought to be a better substitute for phenacetin (Ehrhart *et al.*, 1965 ▸; Ehrhart & Ott, 1958 ▸), bucetin was introduced into the markets in Germany but was soon withdrawn from use because of renal toxicity and risk of carcinogenesis (Fung *et al.*, 2001 ▸; Togei *et al.*, 1987 ▸). The renal toxicity of bucetin, renal papillary necrosis, is similar in nature to that induced by phenacetin but is somewhat less pronounced, presumably due to differences in the rates of de­acyl­ation by microsomal enzymes leading to the formation of 4-eth­oxy­aniline (Nohmi *et al.*, 1984 ▸). Thus, the renal papillary necrosis by phenacetin and bucetin appears to be a manifestation of the formation of 4-eth­oxy­aniline and the subsequent inhibitory action(s) of this putative metabolite (or its hy­droxy­lated and/or autooxidation products, *N*-(4-eth­oxy­phen­yl)hydroxyl­amine and 1-eth­oxy-4-nitroso­benzene) on PGE2 synthesis and a possible reduction of COX-2 expression (Camus *et al.*, 1982 ▸; Goodin *et al.*, 2002 ▸; Kankuri *et al.*, 2003 ▸; Wirth *et al.*, 1982 ▸).

Previous studies from our laboratory and elsewhere have shown that celluar oxidants, such as per­oxy­nitrite/per­oxy­nitrous acid and hypochlorite/hypo­chlorous acid, can constitute an important pathway for non-enzymatic bio­transformation of *N*-(4-hy­droxy­phen­yl)acetamide (Bedner & MacCrehan, 2006 ▸; Uppu & Martin, 2004 ▸; Whiteman *et al.*, 1996 ▸), apocynin (Gernapudi *et al.*, 2009 ▸), clozapine (Frimat *et al.*, 1997 ▸; Uppu *et al.*, 2005 ▸), and certain other xenobiotics (Babu *et al.*, 2012 ▸; Ju & Uetrecht, 1998 ▸; Rattay & Benndorf, 2021 ▸). We believe that the above referenced oxidants may also be involved in the biotransformation of bucetin, leading to the formation of hy­droxy­lated, chlorinated, and nitrated products and thus contribute to the toxicity. To address this and to better understand the mechanisms of toxicity of bucetin and phenacetin and its congeners, we determined the crystal structure of racemic bucetin.

**Figure 1:**
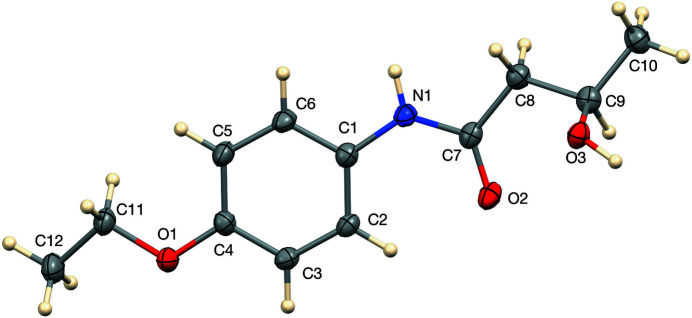
Mol­ecular structure of *N*-(4-eth­oxy­phen­yl)-3-hy­droxy­butanamide with displacement ellipsoids drawn at the 50% probability level.

The mol­ecular structure of the title compound, racemic bucetin, is shown in Fig. 1 ▸. The mol­ecule is in an extended conformation as illustrated by torsion angle C4—O1—C11—C12 [170.14 (15)°] in the eth­oxy group and torsion angles C1—N1—C7—C8 [−177.24 (16)°], N1—C7—C8—C9 [170.08 (15)°] and C7—C8—C9—C10 [171.41 (15)°] in the butanamide chain. In the arbitrarily chosen asymmetric molecule, atom C9 has an *R* configuration, but crystal symmetry generates a racemic mixture.

**Figure 2:**
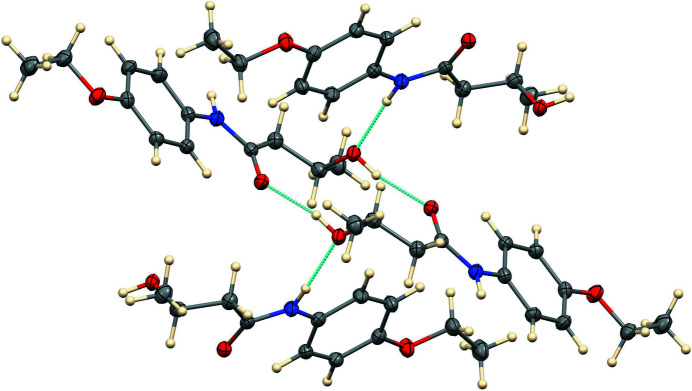
The hydrogen bonding in the packing of *N*-(4-eth­oxy­phen­yl)-3-hy­droxy­butanamide.

**Figure 3:**
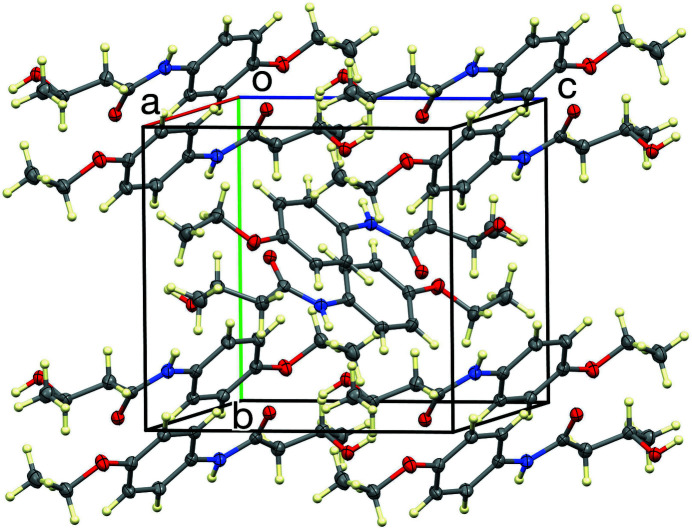
Crystal packing of the title compound *N*-(4-eth­oxy­phen­yl)-3-hy­droxy­butanamide.

As shown in Fig. 2 ▸, the OH group donates an inter­molecular hydrogen bond to the amide carbonyl oxygen atom and accepts an inter­molecular hydrogen bond from an adjacent N—H group. The donor–acceptor separations for these hydrogen bonds are 2.7268 (17) Å for O—H⋯O(−*x* + 1, −*y* + 1, −*z* + 2) and 2.8611 (19) Å for N—H⋯O(*x*, −*y* + 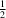 , *z* − 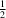 ). The former thus forms 12-membered dimeric rings about inversion centers, and the latter form chains in the [001] direction. The overall hydrogen-bonded network is two-dimensional, with no propagation in the [100] direction. The packing in the unit cell is shown in Fig. 3 ▸ and includes also C—H⋯O inter­actions (Table 1 ▸).

Given the current understanding that de-acyl­ation constitutes an important step in the expression of renal toxicity (Kankuri *et al.*, 2003 ▸; Nohmi *et al.*, 1984 ▸; Taxak *et al.*, 2013 ▸), and the fact that the acyl group in bucetin (3-hy­droxy­butyr­yl) is much larger in size compared to the acetyl group in phenacetin and its congeners and has a chiral center, the information on the crystal structure of bucetin presented here may help in the development of analgesics with little or no renal toxicity.

## Synthesis and crystallization

The title compound, C_12_H_17_NO_3_ (bucetin; CAS No. 1083–57-4) was obtained from Sigma-Aldrich, St. Louis, MO and was used without further purification. Single crystals of racemic bucetin were prepared by slow cooling of a nearly saturated solution of bucetin in boiling deionized water.

## Refinement

Crystal data, data collection and structure refinement details are summarized in Table 2 ▸.

## Supplementary Material

Crystal structure: contains datablock(s) I. DOI: 10.1107/S2414314623002316/vm4059sup1.cif

Structure factors: contains datablock(s) I. DOI: 10.1107/S2414314623002316/vm4059Isup2.hkl

Supporting information file. DOI: 10.1107/S2414314623002316/vm4059Isup3.cml

CCDC reference: 2247342

Additional supporting information:  crystallographic information; 3D view; checkCIF report

## Figures and Tables

**Table 1:** Hydrogen-bond geometry (Å, °)

*D*—H⋯*A*	*D*—H	H⋯*A*	*D*⋯*A*	*D*—H⋯*A*
O3—H3*O*⋯O2^i^	0.89 (2)	1.85 (2)	2.7268 (17)	167 (2)
N1—H1*N*⋯O3^ii^	0.88 (2)	1.99 (2)	2.8611 (19)	169.7 (19)
C2—H2⋯O2	0.95	2.32	2.908 (2)	119
C3—H3*A*⋯O1^iii^	0.95	2.60	3.482 (2)	154
C6—H6⋯O2^ii^	0.95	2.65	3.468 (2)	145
C6—H6⋯O3^ii^	0.95	2.48	3.269 (2)	141
C8—H8*B*⋯O2^iv^	0.99	2.58	3.553 (2)	167

Symmetry codes: (i) 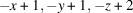 ; (ii) 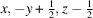 ; (iii) 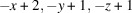 ; (iv) 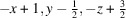 .

**Table 2:** Experimental details

Crystal data	
Chemical formula	C_12_H_17_NO_3_
*M*_r_	223.26
Crystal system, space group	Monoclinic, *P*2_1_/*c*
Temperature (K)	100
*a*, *b*, *c* (Å)	12.2343 (4), 9.6404 (3), 9.9098 (3)
β (°)	93.295 (2)
*V* (Å^3^)	1166.86 (6)
*Z*	4
Radiation type	Cu *K*α
μ (mm^−1^)	0.75
Crystal size (mm)	0.14 × 0.14 × 0.01

Data collection	
Diffractometer	Bruker Kappa APEXII CCD DUO
Absorption correction	Multi-scan (*SADABS*; Krause *et al.*, 2015 ▸)
*T*_min_, *T*_max_	0.832, 0.993
No. of measured, independent and observed [*I* > 2σ(*I*)] reflections	14229, 2139, 1739
*R*_int_	0.061
(sin θ/λ)_max_ (Å^−1^)	0.603

Refinement	
*R*[*F* ^2^ > 2σ(*F* ^2^)], *wR*(*F* ^2^), *S*	0.045, 0.116, 1.06
No. of reflections	2139
No. of parameters	153
H-atom treatment	H atoms treated by a mixture of independent and constrained refinement
Δρ_max_, Δρ_min_ (e Å^−3^)	0.40, −0.23

Computer programs: *APEX2* and *SAINT* (Bruker, 2016 ▸), *SHELXT2014/5* (Sheldrick, 2015*a* ▸), *SHELXL2017/1* (Sheldrick, 2015*b* ▸), *Mercury* (Macrae *et al.*, 2020 ▸), and *publCIF* (Westrip, 2010 ▸).

## full crystallographic data

### Crystal data

**Table:**

C_12_H_17_NO_3_	*F*(000) = 480
*M**_r_* = 223.26	*D*_x_ = 1.271 Mg m^−^^3^
Monoclinic, *P*2_1_/*c*	Cu *K*α radiation, λ = 1.54184 Å
*a* = 12.2343 (4) Å	Cell parameters from 3666 reflections
*b* = 9.6404 (3) Å	θ = 3.6–68.5°
*c* = 9.9098 (3) Å	µ = 0.75 mm^−^^1^
β = 93.295 (2)°	*T* = 100 K
*V* = 1166.86 (6) Å^3^	Plate, colourless
*Z* = 4	0.14 × 0.14 × 0.01 mm

### Data collection

**Table:**

Bruker Kappa APEXII CCD DUO diffractometer	2139 independent reflections
Radiation source: IµS microfocus	1739 reflections with *I* > 2σ(*I*)
QUAZAR multilayer optics monochromator	*R*_int_ = 0.061
φ and ω scans	θ_max_ = 68.5°, θ_min_ = 3.6°
Absorption correction: multi-scan (SADABS; Krause *et al.*, 2015)	*h* = −14→14
*T*_min_ = 0.832, *T*_max_ = 0.993	*k* = −11→11
14229 measured reflections	*l* = −11→11

### Refinement

**Table:**

Refinement on *F*^2^	0 restraints
Least-squares matrix: full	Hydrogen site location: mixed
*R*[*F*^2^ > 2σ(*F*^2^)] = 0.045	H atoms treated by a mixture of independent and constrained refinement
*wR*(*F*^2^) = 0.116	*w* = 1/[σ^2^(*F*_o_^2^) + (0.0542*P*)^2^ + 0.4983*P*] where *P* = (*F*_o_^2^ + 2*F*_c_^2^)/3
*S* = 1.06	(Δ/σ)_max_ < 0.001
2139 reflections	Δρ_max_ = 0.40 e Å^−^^3^
153 parameters	Δρ_min_ = −0.23 e Å^−^^3^

### Special details

**Table:**

Geometry. All esds (except the esd in the dihedral angle between two l.s. planes) are estimated using the full covariance matrix. The cell esds are taken into account individually in the estimation of esds in distances, angles and torsion angles; correlations between esds in cell parameters are only used when they are defined by crystal symmetry. An approximate (isotropic) treatment of cell esds is used for estimating esds involving l.s. planes.
Refinement. All H atoms were located in difference maps and those on C were thereafter treated as riding in geometrically idealized positions with C—H distances 0.95 Å for phenyl, 0.98 Å for methyl, 0.99 Å for CH_2_, and 1.00 Å for methine. Coordinates of the N—H and O—H hydrogen atoms were refined. *U*_iso_(H) values were assigned as 1.2*U*_eq_ for the attached atom (1.5 for methyl and OH).

### Fractional atomic coordinates and isotropic or equivalent isotropic displacement parameters (Å^2^)

**Table:**

	*x*	*y*	*z*	*U*_iso_*/*U*_eq_	
O1	0.93903 (10)	0.38529 (13)	0.33811 (12)	0.0251 (3)	
O2	0.56520 (10)	0.51646 (12)	0.76720 (12)	0.0250 (3)	
O3	0.43094 (10)	0.37235 (13)	0.97956 (12)	0.0225 (3)	
H3O	0.4299 (17)	0.421 (2)	1.056 (2)	0.034*	
N1	0.54676 (12)	0.35963 (15)	0.59755 (15)	0.0214 (3)	
H1N	0.5043 (17)	0.295 (2)	0.559 (2)	0.026*	
C1	0.64870 (14)	0.36976 (17)	0.53789 (17)	0.0203 (4)	
C2	0.73038 (14)	0.46664 (17)	0.57328 (17)	0.0204 (4)	
H2	0.720136	0.531692	0.643445	0.024*	
C3	0.82674 (14)	0.46737 (17)	0.50532 (17)	0.0214 (4)	
H3A	0.882226	0.533130	0.529774	0.026*	
C4	0.84298 (14)	0.37343 (18)	0.40232 (17)	0.0211 (4)	
C5	0.76284 (15)	0.27562 (18)	0.36878 (18)	0.0247 (4)	
H5	0.773553	0.209619	0.299592	0.030*	
C6	0.66716 (15)	0.27482 (18)	0.43681 (18)	0.0236 (4)	
H6	0.612697	0.207359	0.413541	0.028*	
C7	0.51039 (14)	0.42701 (17)	0.70506 (17)	0.0204 (4)	
C8	0.39683 (15)	0.38164 (18)	0.74180 (18)	0.0238 (4)	
H8A	0.342927	0.412240	0.669554	0.029*	
H8B	0.394699	0.279044	0.744956	0.029*	
C9	0.36269 (15)	0.43784 (18)	0.87556 (18)	0.0237 (4)	
H9	0.375158	0.540341	0.878649	0.028*	
C10	0.24341 (15)	0.4077 (2)	0.8973 (2)	0.0291 (4)	
H10A	0.231907	0.307161	0.899454	0.044*	
H10B	0.197425	0.448185	0.823174	0.044*	
H10C	0.223748	0.448333	0.983239	0.044*	
C11	0.95150 (15)	0.29641 (19)	0.22356 (19)	0.0267 (4)	
H11A	0.886016	0.302629	0.160352	0.032*	
H11B	0.960301	0.198852	0.253434	0.032*	
C12	1.05153 (16)	0.3435 (2)	0.1548 (2)	0.0336 (5)	
H12A	1.042081	0.440295	0.126142	0.050*	
H12B	1.061736	0.285085	0.075633	0.050*	
H12C	1.115910	0.335942	0.217943	0.050*	

### Atomic displacement parameters (Å^2^)

**Table:**

	*U*^11^	*U*^22^	*U*^33^	*U*^12^	*U*^13^	*U*^23^
O1	0.0241 (7)	0.0297 (7)	0.0221 (7)	−0.0038 (5)	0.0063 (5)	−0.0055 (5)
O2	0.0345 (7)	0.0209 (7)	0.0204 (6)	−0.0026 (5)	0.0082 (5)	−0.0018 (5)
O3	0.0279 (7)	0.0242 (7)	0.0153 (6)	0.0032 (5)	0.0013 (5)	0.0005 (5)
N1	0.0241 (8)	0.0217 (8)	0.0186 (8)	−0.0041 (6)	0.0036 (6)	−0.0017 (6)
C1	0.0244 (9)	0.0196 (9)	0.0171 (9)	−0.0003 (6)	0.0026 (7)	0.0022 (6)
C2	0.0266 (9)	0.0177 (8)	0.0168 (8)	−0.0010 (7)	0.0020 (7)	−0.0006 (6)
C3	0.0250 (9)	0.0204 (9)	0.0188 (9)	−0.0035 (7)	0.0004 (7)	0.0002 (6)
C4	0.0217 (9)	0.0241 (9)	0.0177 (9)	0.0000 (7)	0.0029 (7)	0.0025 (7)
C5	0.0302 (10)	0.0227 (9)	0.0219 (9)	−0.0027 (7)	0.0070 (7)	−0.0054 (7)
C6	0.0282 (9)	0.0209 (9)	0.0222 (9)	−0.0070 (7)	0.0050 (7)	−0.0032 (7)
C7	0.0280 (9)	0.0167 (8)	0.0168 (8)	0.0024 (7)	0.0024 (7)	0.0025 (7)
C8	0.0261 (10)	0.0247 (9)	0.0208 (9)	−0.0002 (7)	0.0016 (7)	0.0012 (7)
C9	0.0272 (10)	0.0220 (9)	0.0220 (9)	0.0025 (7)	0.0024 (7)	0.0029 (7)
C10	0.0267 (10)	0.0327 (10)	0.0283 (10)	0.0003 (8)	0.0053 (8)	0.0033 (8)
C11	0.0304 (10)	0.0260 (10)	0.0245 (10)	0.0005 (7)	0.0082 (8)	−0.0048 (7)
C12	0.0330 (11)	0.0381 (11)	0.0312 (11)	−0.0024 (8)	0.0135 (9)	−0.0070 (8)

### Geometric parameters (Å, º)

**Table:**

O1—C4	1.373 (2)	C6—H6	0.9500
O1—C11	1.437 (2)	C7—C8	1.521 (2)
O2—C7	1.235 (2)	C8—C9	1.513 (2)
O3—C9	1.435 (2)	C8—H8A	0.9900
O3—H3O	0.89 (2)	C8—H8B	0.9900
N1—C7	1.345 (2)	C9—C10	1.515 (3)
N1—C1	1.414 (2)	C9—H9	1.0000
N1—H1N	0.88 (2)	C10—H10A	0.9800
C1—C6	1.385 (2)	C10—H10B	0.9800
C1—C2	1.398 (2)	C10—H10C	0.9800
C2—C3	1.391 (2)	C11—C12	1.505 (3)
C2—H2	0.9500	C11—H11A	0.9900
C3—C4	1.387 (2)	C11—H11B	0.9900
C3—H3A	0.9500	C12—H12A	0.9800
C4—C5	1.387 (3)	C12—H12B	0.9800
C5—C6	1.384 (2)	C12—H12C	0.9800
C5—H5	0.9500		
			
C4—O1—C11	116.67 (13)	C7—C8—H8A	108.7
C9—O3—H3O	110.2 (14)	C9—C8—H8B	108.7
C7—N1—C1	129.62 (15)	C7—C8—H8B	108.7
C7—N1—H1N	118.0 (14)	H8A—C8—H8B	107.6
C1—N1—H1N	112.2 (14)	O3—C9—C8	107.05 (14)
C6—C1—C2	118.62 (16)	O3—C9—C10	109.82 (15)
C6—C1—N1	116.18 (15)	C8—C9—C10	111.86 (15)
C2—C1—N1	125.20 (16)	O3—C9—H9	109.4
C3—C2—C1	119.71 (16)	C8—C9—H9	109.4
C3—C2—H2	120.1	C10—C9—H9	109.4
C1—C2—H2	120.1	C9—C10—H10A	109.5
C4—C3—C2	120.94 (16)	C9—C10—H10B	109.5
C4—C3—H3A	119.5	H10A—C10—H10B	109.5
C2—C3—H3A	119.5	C9—C10—H10C	109.5
O1—C4—C5	123.90 (16)	H10A—C10—H10C	109.5
O1—C4—C3	116.69 (15)	H10B—C10—H10C	109.5
C5—C4—C3	119.40 (16)	O1—C11—C12	107.67 (15)
C6—C5—C4	119.55 (16)	O1—C11—H11A	110.2
C6—C5—H5	120.2	C12—C11—H11A	110.2
C4—C5—H5	120.2	O1—C11—H11B	110.2
C5—C6—C1	121.75 (16)	C12—C11—H11B	110.2
C5—C6—H6	119.1	H11A—C11—H11B	108.5
C1—C6—H6	119.1	C11—C12—H12A	109.5
O2—C7—N1	122.49 (16)	C11—C12—H12B	109.5
O2—C7—C8	123.98 (15)	H12A—C12—H12B	109.5
N1—C7—C8	113.52 (15)	C11—C12—H12C	109.5
C9—C8—C7	114.13 (15)	H12A—C12—H12C	109.5
C9—C8—H8A	108.7	H12B—C12—H12C	109.5
			
C7—N1—C1—C6	172.74 (17)	C4—C5—C6—C1	0.2 (3)
C7—N1—C1—C2	−7.1 (3)	C2—C1—C6—C5	−1.4 (3)
C6—C1—C2—C3	1.1 (2)	N1—C1—C6—C5	178.76 (16)
N1—C1—C2—C3	−178.98 (16)	C1—N1—C7—O2	2.5 (3)
C1—C2—C3—C4	0.2 (3)	C1—N1—C7—C8	−177.24 (16)
C11—O1—C4—C5	4.6 (2)	O2—C7—C8—C9	−9.6 (2)
C11—O1—C4—C3	−174.53 (15)	N1—C7—C8—C9	170.08 (15)
C2—C3—C4—O1	177.82 (15)	C7—C8—C9—O3	−68.25 (18)
C2—C3—C4—C5	−1.4 (3)	C7—C8—C9—C10	171.41 (15)
O1—C4—C5—C6	−177.96 (16)	C4—O1—C11—C12	170.14 (15)
C3—C4—C5—C6	1.2 (3)		

### Hydrogen-bond geometry (Å, º)

**Table:**

*D*—H···*A*	*D*—H	H···*A*	*D*···*A*	*D*—H···*A*
O3—H3*O*···O2^i^	0.89 (2)	1.85 (2)	2.7268 (17)	167 (2)
N1—H1*N*···O3^ii^	0.88 (2)	1.99 (2)	2.8611 (19)	169.7 (19)
C2—H2···O2	0.95	2.32	2.908 (2)	119
C3—H3*A*···O1^iii^	0.95	2.60	3.482 (2)	154
C6—H6···O2^ii^	0.95	2.65	3.468 (2)	145
C6—H6···O3^ii^	0.95	2.48	3.269 (2)	141
C8—H8*B*···O2^iv^	0.99	2.58	3.553 (2)	167

Symmetry codes: (i) −*x*+1, −*y*+1, −*z*+2; (ii) *x*, −*y*+1/2, *z*−1/2; (iii) −*x*+2, −*y*+1, −*z*+1; (iv) −*x*+1, *y*−1/2, −*z*+3/2.

## References

[bb1] Babu, S., Vellore, N. A., Kasibotla, A. V., Dwayne, H. J., Stubblefield, M. A. & Uppu, R. M. (2012). *Biochem. Biophys. Res. Commun.* **426**, 215–220. 10.1016/j.bbrc.2012.08.065 22935422

[bb2] Bedner, M. & MacCrehan, W. A. (2006). *Environ. Sci. Technol.* **40**, 516–522. 10.1021/es0509073 16468397

[bb3] Bruker (2016). *APEX2* and *SAINT*. Bruker AXS Inc., Madison, Wisconsin, USA.

[bb4] Camus, A. M., Friesen, M., Croisy, A. & Bartsch, H. (1982). *Cancer Res.* **42**, 3201–3208. 7046920

[bb5] Ehrhart, G., Lindner, E. & Häussler, A. (1965). *Arzneimittelforschung*, **15**, 727–738. 5898671

[bb6] Ehrhart, G. & Ott, H. (1958). US Patent 2830087

[bb7] Frimat, B., Gressier, B., Odou, P., Brunet, C., Dine, T., Luycky, M., Cazin, M. & Cazin, J. C. (1997). *Fundam. Clin. Pharmacol.* **11**, 267–274. 10.1111/j.1472-8206.1997.tb00195.x 9243259

[bb8] Fung, M., Thornton, A., Mybeck, K., Wu, J. H. H., Hornbuckle, K. & Muniz, E. (2001). *Ther. Innov. Regul. Sci*, **35**, 293–317.

[bb9] Gernapudi, R., Babu, S., Raghavamenon, A. C. & Uppu, R. M. (2009). *FASEB J.* **23** (S1), LB397.

[bb10] Goodin, M. G., Walker, R. J. & Rosengren, R. J. (2002). *Res. Commun. Mol. Pathol. Pharmacol.* **111**, 153–166. 14632321

[bb11] Ju, C. & Uetrecht, J. P. (1998). *Drug Metab. Dispos.* **26**, 676–680. 9660850

[bb12] Kankuri, E., Solatunturi, E. & Vapaatalo, H. (2003). *Thromb. Res.* **110**, 299–303. 10.1016/s0049-3848(03)00416-x 14592552

[bb13] Krause, L., Herbst-Irmer, R., Sheldrick, G. M. & Stalke, D. (2015). *J. Appl. Cryst.* **48**, 3–10. 10.1107/S1600576714022985 PMC4453166 26089746

[bb14] Macrae, C. F., Sovago, I., Cottrell, S. J., Galek, P. T. A., McCabe, P., Pidcock, E., Platings, M., Shields, G. P., Stevens, J. S., Towler, M. & Wood, P. A. (2020). *J. Appl. Cryst.* **53**, 226–235. 10.1107/S1600576719014092 PMC6998782 32047413

[bb15] Nohmi, T., Yoshikawa, K., Ishidate, M. Jr, Hiratsuka, A. & Watabe, T. (1984). *Chem. Pharm. Bull.* **32**, 4525–4531. 10.1248/cpb.32.4525 6398128

[bb16] Rattay, B. & Benndorf, R. A. (2021). *Front. Pharamcol.* **12**, x727717. 10.3389/fphar.2021.727717 PMC8414253 34483939

[bb17] Sheldrick, G. M. (2015*a*). *Acta Cryst.* A**71**, 3–8.

[bb18] Sheldrick, G. M. (2015*b*). *Acta Cryst.* C**71**, 3–8.

[bb19] Taxak, N., Chaitanya Prasad, K. & Bharatam, P. V. (2013). *Comput. Theor. Chem.* **1007**, 48–56.

[bb20] Togei, K., Sano, N., Maeda, T., Shibata, M. & Otsuka, H. (1987). *J. Natl Cancer Inst.* **79**, 1151–1158. 3479641

[bb21] Uppu, R. M. & Martin, R. J. (2004). *The Toxicologist* (supplement to *Toxicol. Sci.*) **84**, 319.

[bb22] Uppu, R. M., Sathishkumar, K. & Perumal, T. E. (2005). Free Radic. Biol. Med. 39 (S1), S15.

[bb23] Westrip, S. P. (2010). *J. Appl. Cryst.* **43**, 920–925.

[bb24] Whiteman, M., Kaur, H. & Halliwell, B. (1996). *Ann. Rheum. Dis.* **55**, 383–387. 10.1136/ard.55.6.383 PMC1010190 8694578

[bb25] Wirth, P. J., Alewood, P., Calder, I. & Thorgeirsson, S. S. (1982). *Carcinogenesis*, **3**, 167–170. 10.1093/carcin/3.2.167 6896016

